# Increasing the Hydrophobic Component of Poloxamers and the Inclusion of Salt Extend the Release of Bupivacaine from Injectable In Situ Gels, While Common Polymer Additives Have Little Effect

**DOI:** 10.3390/gels8080484

**Published:** 2022-08-02

**Authors:** Hani Abdeltawab, Darren Svirskis, Andrew G. Hill, Manisha Sharma

**Affiliations:** 1 School of Pharmacy, Faculty of Medical & Health Sciences, The University of Auckland, Auckland 1023, New Zealand; h.abdeltawab@auckland.ac.nz (H.A.); d.svirskis@auckland.ac.nz (D.S.); 2 Department of Surgery, South Auckland Clinical Campus, The University of Auckland, Middlemore Hospital, Auckland 2025, New Zealand; a.hill@auckland.ac.nz

**Keywords:** poloxamers, bupivacaine hydrochloride, sustained drug release, burst release, additive polymer, physical blending, sodium chloride

## Abstract

Various strategies have been applied to reduce the initial burst of drug release and sustain release from poloxamer-based thermoresponsive gels. This work focussed on investigating different formulation approaches to minimise the initial burst of release and sustain the release of the small hydrophilic drug bupivacaine hydrochloride from poloxamer-based thermoresponsive gels. Various in situ gel formulations were prepared by varying the polypropylene oxide (PPO)/polyethylene oxide (PEO) ratio and by adding additives previously described in the literature. It was observed that increasing the PPO/PEO ratio from 0.28 to 0.30 reduced the initial burst release from 17.3% ± 1.8 to 9.1% ± 1.2 during the first six hours and extended the release profile from 10 to 14 days. Notably, the inclusion of sodium chloride (NaCl 0.4% *w*/*w*) further reduced the initial burst release to 1.8% ± 1.1 over the first 6 h. Meanwhile, physical blending with additive polymers had a negligible effect on the burst release and overall release profile. The findings suggest that extended release of bupivacaine hydrochloride, with reduced initial burst release, can be achieved simply by increasing the PPO/PEO ratio and the inclusion of NaCl.

## 1. Introduction

Poloxamers are Food and Drug Administration (FDA)-approved triblock copolymers, composed of a central hydrophobic block of polypropylene oxide (PPO) and two hydrophilic terminals of polyethylene oxide (PEO) [1,2]. They are increasingly used in drug delivery, due to their favourable properties and versatility. Their concentrated solutions are liquids at ambient temperature, allowing parenteral administration, whereas they form a biocompatible and bioerodible gel at physiological temperatures [3,4]. In addition, attributed to their amphiphilic properties, they can deliver both hydrophilic and hydrophobic drugs [1]. In this manuscript, we explore approaches to alter drug release behaviour from poloxamers in order to support formulations, to be tuned to fit a range of applications.

The use of poloxamers as sustained delivery platforms is challenged by their rapid erosion and high rates of drug diffusion. This is attributed to their poor mechanical properties and continuous interconnected aqueous channels within the gel matrix, causing a rapid release of the loaded drug [1,5,6,7]. In addition, many of the described poloxamer-based gels exhibit an initial burst release of the loaded drug [8,9,10,11], attributed to the lag time between administration and phase transition. These attributes raise concerns around the safety and efficacy of poloxamer-based thermoresponsive gel systems for drug delivery [12].

Many studies report that blending of additive polymers, such as alginates [13,14], cellulose derivatives [15,16], and chitosan [17,18], with poloxamers extends the release profile of the loaded drug. These findings can be explained by the enhanced mechanical and rheological properties of poloxamer-based in situ gels, which have been widely reported with the addition of the above-mentioned polymers. However, there are contradictory reports of carboxymethyl cellulose sodium (4% *w*/*w*) leading to an increase in the rate of drug release [19]. Meanwhile, blending of hydroxyethyl cellulose (2% *w*/*w*), hydroxypropyl methylcellulose (0.5% *w*/*w*), and polyethylene glycol 400 (5% *w*/*w*) into poloxamers did not alter the release profile of loaded drugs [14,20,21].

Similarly, the influence of sodium chloride (NaCl) on drug release from poloxamer gels is debatable. While it has been widely demonstrated that the inclusion of NaCl significantly enhances the gel mechanical and rheological properties [1], its effect on drug release is not fully understood. It has been reported that NaCl (1% *w*/*w*) increased the percentage of meloxicam (MW = 341.5, practically water insoluble) released over six hours from 75% to 80% [21]. The authors explained that NaCl diffuses quickly into the release medium owing to its high aqueous solubility, leading to the formation of a highly porous matrix. On the other hand, it has been reported that NaCl (1% *w*/*w*) reduced the percentage of nimesulide (MW = 308, sparingly water soluble) released in 7 h from 90% to 70% [14]. Likewise, NaCl (2% *w*/*w*) reduced the diffusion coefficient of atrial natriuretic factor (a natriuretic peptide hormone, MW = 1225.4) from poloxamer 407 at 37 °C [22]. This reduction in diffusion coefficient is thought to be due to the associated increase in gel rheological properties. On the other hand, the inclusion of NaCl (0.8% *w*/*w*) did not alter the release of diclofenac sodium (MW = 318.1, poorly water soluble) from a thermoresponsive gel system prepared using different poloxamers [23].

The release of drugs from poloxamer thermoresponsive gels is significantly affected by the hydrophobicity/hydrophilicity (PPO/PEO) balance of the gel matrix. It has been widely demonstrated that increasing the hydrophobic content enhances micellar dehydration and entanglement. This in turn produces less porous and stronger gels, retarding the release of the loaded drug [1]. On the contrary, it has been demonstrated that reducing the PPO/PEO ratio via blending the more hydrophilic poloxamer (P188) relative to the more hydrophobic poloxamer (P407) increases the release of loaded drugs: meloxicam, diclofenac sodium, and nonoxynol-9 [21,23,24]. However, in these studies, the total poloxamer concentration was not considered, which is known to influence drug release rates.

The selection of a suitable formulation strategy to achieve an extended-release profile also requires critical consideration of the intended application. For example, the viscosity of poloxamer-based thermoresponsive gels for parenteral administration must be considered. Blending of additive polymers can significantly increase the viscosity of poloxamer thermoresponsive gels, causing difficulty in the syringeability/injectability of the developed formulation [25]. In addition, too high PPO/PEO ratio or inclusion of NaCl can alter the phase transition, rendering the formulations unsuitable for the intended application [23,26].

**Figure 1:**
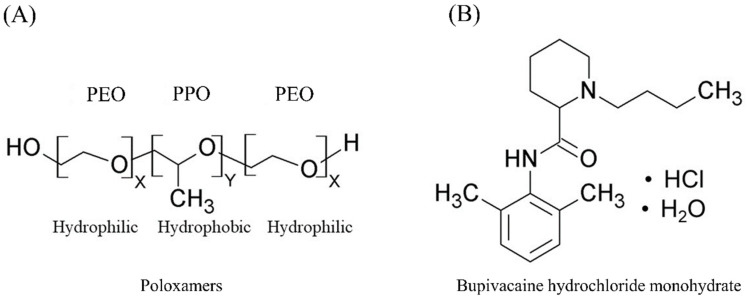
Chemical structure of (**A**) poloxamers, where X and Y are the average numbers of repeating units of each blockchain (X is 76 and Y is 29 for poloxamer 188, while X is 100 and Y is 65 for poloxamer 407) and (**B**) bupivacaine hydrochloride monohydrate (BH).

This work aimed to investigate commonly used formulation strategies, namely physical blending with additive polymers or NaCl and altering PPO/PEO ratio, to achieve extended-release profiles from poloxamer-based thermoresponsive gels. Bupivacaine hydrochloride (BH, Figure 1B) was chosen as a model drug in this study. BH is a long-acting local anaesthetic agent, commonly used in postoperative pain management [27]. It has a molecular weight of 342.9 with an aqueous solubility (40–50 mg/mL) in the pH range of 1–6 and exhibits lower solubility (26 mg/mL) in phosphate-buffered saline (pH 7.4) [28]. The study aimed to compare the effect of the above-mentioned formulation strategies, to provide researchers with recommendations on the choice of a formulation approach to tailor drug release from poloxamer-based in situ gelling systems.

## 2. Materials and Methods

### 2.1. Materials

Bupivacaine hydrochloride monohydrate (BH) was purchased from Jai Radhe Sales (Gujarat, India). Poloxamer 407 (P407), poloxamer 188 (P188), and phosphate-buffered saline (PBS) tablets were sourced from Sigma-Aldrich (St. Louis, MO, USA). Sodium chloride, sodium alginate (medium viscosity), and methylcellulose (454.5 g/mol) were sourced from Sigma-Aldrich (Gillingham, UK). Chitosan (medium molecular weight) was purchased from Sigma-Aldrich (Iceland), and carboxymethylcellulose (263.2 g/mol) was purchased from VWR International Ltd., (Poole, UK). Water was obtained from a Milli-Q water purification system (Millipore, Darmstadt, Germany) with an 18 MΩ.cm resistivity. All other solvents and reagents used were of analytical grade.

### 2.2. Preparation and Optimisation of Thermoresponsive Gel Formulations

Thermoresponsive gels were prepared by the cold method [10]. Table 1 gives the details of all the formulations trialled. Briefly, specified weights of P407 and P188 were dissolved in Milli-Q water at 4 °C, by stirring overnight. Various concentrations of P407 (namely, 19, 21, 23, 25, and 27% *w*/*w*) were mixed with a constant P188 concentration (5.5% *w*/*w*), and the developed formulations were screened for their sol-to-gel transition temperatures using a tube inversion method [11]. The formulation with the highest PPO/PEO ratio (0.3:1) containing 23% *w*/*w* P407 and 5.5% *w*/*w* P188, which maintained its liquid status at room temperature, was chosen as the base formulation in this project. To demonstrate the influence of the PPO/PEO ratio, a formulation with the same total poloxamer concentration and different PPO/PEO ratio, as compared to the base formulation, was prepared (20% *w*/*w* P407 and 8.5% *w*/*w* P188). To study the effect of additives, the base formulation was blended with varied concentrations of selected additives: sodium alginate (0.5, 0.75, and 1% *w*/*w*), methylcellulose (0.35 and 0.75% *w*/*w*), carboxymethylcellulose (0.35, 0.5, and 0.75% *w*/*w*), chitosan (0.4, 0.5, and 0.65% *w*/*w*), and sodium chloride (0.4, 0.6, and 0.9% *w*/*w*). Formulations with suitable sol-to-gel transition temperatures (25–37 °C), were loaded with BH (1% *w*/*w*) and underwent further characterisation.

The tube inversion method was employed to identify the sol-to-gel transition temperature [11]. Briefly, 1.5 mL of each formulation was transferred into a 2 mL Eppendorf tube and left for 5 min at room temperature for equilibration. The tubes were then transferred to a thermomixer (Eppendorf, Hamburg, Germany), previously equilibrated at 20 °C, and subjected to a temperature increment of 1 °C each 2 min. The sample was considered gelled if 90° movement of the Eppendorf tube resulted in no movement of the meniscus [11].

The sol-to-gel transition time was determined by transferring 1.5 mL of formulation into a 2 mL Eppendorf tube and immediately placed in a thermomixer previously set at 37 °C. The time required to achieve sol-to-gel transition was recorded. All experiments were conducted in triplicate.

### 2.3. Fourier Transform Infra-Red Spectroscopy (FTIR)

FTIR analysis was performed to study the intermolecular interactions between poloxamers and BH, and poloxamers and additives. Briefly, FTIR spectra were obtained for the freeze-dried powders of the blank base formulation (F1), BH, their 10:1 (*w*/*w*) mixture, and selected blank formulations with additives, namely, F1NaCl, F1CH, F1MC, F1CMC, and F1SA. All FTIR measurements were conducted in attenuated total reflectance (ATR) mode, using a Bruker Tensor 37 FTIR spectrophotometer (Bruker Tensor 37, Ettlingen, Germany). Samples were scanned over the range of 400–4000 cm^−1^ wavenumbers with a spectral resolution of 1 cm^−1^ and 64 scans per sample. The spectral data were analysed using Bruker OPUS software version 8.5 (SP1) (Bruker Optics GmbH, Ettlingen, Germany).

### 2.4. Mechanical Properties

#### 2.4.1. Gel Strength

A texture analyser (TA.XT Stable Microsystems, Surrey, UK) was employed to study the gel strength of the developed gel formulations [29]. Briefly, a 20 mL cylindrical glass vial (25 mm internal diameter) containing the respective gel formulation (15 mL) was incubated at 37 °C using a temperature-controlled Peltier cabinet for 20 min to ensure complete gel formation. Following the formation of a gel, a 10 mm diameter Delrin cylinder probe was allowed to penetrate the gel to a depth of 5 mm, at a trigger force of 5.0 g, and at a speed of 1 mm/s. A typical force–time curve was used to determine the gel strength and hardness of the respective formulations by using the Exponent 32 software version 6 (TA.XT Stable Microsystems, Surrey, UK) [30]. All experiments were carried out in triplicate.

#### 2.4.2. Injectability

A texture analyser equipped with a universal syringe rig attachment (A/USR, Stable Microsystems, Surrey, UK) was used to study the injectability of the gel formulations. The method was adopted from Shavandi et al., with slight modifications [31]. A 3 mL syringe (18-gauge needle) was filled with the formulation and attached to the universal syringe rig. The texture analyser probe was then used to push the syringe plunger to a distance of 20 mm at a speed of 5 mm/s. The injectability was then determined by measuring the maximum force and the total work required to expel the formulation out of the syringe through the 18-gauge needle [29,32].

### 2.5. Rheological Properties

An advanced rheometer (AR-G2, TA instruments, Melbourne, Australia) was used to study the rheological behaviour of the developed formulations at two temperatures: 20 °C and 37 °C. All experiments were carried out using a stainless-steel parallel plate geometry (40 mm) and a temperature-controlled Peltier plate, in triplicate.

#### 2.5.1. Frequency Sweep

First, the linear viscoelastic region of the gel formulations was identified by carrying out a strain-sweep analysis at 37 °C ± 0.1 over a wide range of strain amplitude (0.01 to 100%) and at fixed angular frequencies (0.1, 10, and 100 rad/s). Then, the viscoelastic behaviour of the gel was studied by performing an oscillation sweep at 37 °C ± 0.1 over an angular frequency range of 0.1 to 100 rad/s at a constant and low strain amplitude (0.02 Pa). The changes in storage and loss moduli were determined as a function of angular frequency [33].

#### 2.5.2. Flow Sweep

The flow properties of the formulations were studied at 20 °C. The change in formulation viscosity as a function of shear rate over the range of 2–200 s^−1^ was monitored.

### 2.6. Development and Validation of an Analytical Method for the Determination of BH

A reverse-phase high-performance liquid chromatography (RP-HPLC) method was developed and validated as per International Conference for Harmonization (ICH) guidelines [34] for linearity, range, accuracy, specificity, precision, the limit of quantification (LOQ), and limit of detection (LOD).

### 2.7. In Vitro Drug Release Studies

In vitro BH release studies were performed under sink conditions using PBS (pH 7.4) [10]. PBS (prewarmed to 37 °C, 4 mL) was placed in a flat bottom Falcon tube, to which a known amount of formulation (1 g) was injected, which immediately formed gel. Sink conditions were determined based on saturation solubility study results conducted in the laboratory. BH concentration never exceeded 1 mg/mL, which is lower than 5% of the saturation solubility (26 mg/mL) at any time. The tubes were then placed on a rocking shaker (adjusted at 10 rpm), placed inside an incubator maintained at 37 °C. At specified time points, aliquots (50 µL) were withdrawn and replaced by an equal volume of fresh prewarmed PBS. The aliquots were appropriately diluted, filtered through 20 µm syringe filters, and the BH concentration was determined using the developed HPLC analytical method. Curves of the cumulative percentage of drug release against time were constructed, and the similarity factor (f2) was determined among the obtained release profiles.

### 2.8. Mathematical Modelling of the Release Profiles

To determine the mechanism of drug release from the developed thermoresponsive gels, kinetic release models, zero order, first order, Higuchi, and Hixson–Crowell, were applied to in vitro drug release data [8,21], and the best fit was determined.

### 2.9. Statistical Analysis of Data

All results are represented as mean ± standard deviation (SD), where n = 3. The statistical differences were determined by performing either a *t*-test or one-way analysis of variance (one-way ANOVA), with multiple comparisons to F1, whichever was applicable. The significance of the difference was calculated using the software GraphPad Prism 8.2.1 (GraphPad Software Inc., San Diego, CA, USA) at a 95% confidence level (*p* ≤ 0.05 indicated statistical significance), unless specified.

## 3. Results and Discussion

### 3.1. Formulation Preparation and Optimisation for Sol-To-Gel Transition Temperature

After screening a wide range of formulations (Table 1), the sol-to-gel transition temperature and time of selected formulations (sol-to-gel transitions between 25 and 37 °C) are reported in Table 2, with and without drug loaded. The desired temperature range (25–37 °C) was selected to support formulation administration through a needle, whereupon it would gel at physiological temperature, with the potential to sustain drug release [1]. Formulation F1 was selected as the base formulation, as it is the formulation with the highest PPO/PEO ratio (0.30), which showed sol-to-gel transition within the desired temperature range.

Formulation F2 was prepared to exclusively study the effect of the PPO/PEO ratio on the gel characteristics, and the total poloxamer concentration was kept consistent with the base formulation F1. As shown, reducing the PPO/PEO ratio from 0.30 to 0.28 significantly (*p* < 0.0001) increased the sol-to-gel transition temperature and time. This is attributed to the abundant hydrogen bonds between the relatively hydrophilic PEO blocks and water, increasing the energy required to break down the hydrogen bonds between PEO blocks and water and, therefore, increasing the sol-to-gel transition temperature [23,29,35,36].

When selected additives (polymers and salt) were blended with the base formulation (F1) at various concentrations, the sol-to-gel transition temperature was reduced (Table 1) as a function of their concentration [13]. This can be attributed to the hydrogen bonding and physical entanglement with poloxamers facilitating the transition to a 3D network [13,37,38,39,40]. Likewise, the inclusion of NaCl, in formulation F1NaCl, significantly (*p* < 0.0001) reduced both the sol-to-gel transition temperature and time as a function of its concentration. This is attributed to the salting-out effect of NaCl, reducing the number of water molecules available for poloxamers and therefore enhancing the hydrophobic interactions among poloxamer chains. This, in turn, reduces the energy required to dehydrate poloxamers, facilitating the sol-to-gel transition at a lower temperature. In addition, NaCl–poloxamer crosslinking facilitates the physical entanglement between micelles, easing the phase transition and causing a reduction in the sol-to-gel transition temperature [23,41,42,43].

Interestingly, BH loading increased the transition temperature of all formulations by almost 1 °C (Table 2), implying a potential BH–poloxamer interaction [44]. The pH of BH-loaded base formulation (F1) was 6.22, which is ≥2 lower than pKa of BH (pKa = 8.15 [28]), suggesting the presence of its ionised form in solution, with potential interaction with the hydrated micellar corona [40,44,45]. The BH–poloxamer interaction was further confirmed from the FTIR results discussed in Section 3.2 below.

The time required to achieve sol-to-gel transition is crucial to predict the in vivo performance. Formulations that exhibit slow phase transition are likely to demonstrate high initial burst release due to the long lag time between administration and transit to a gel form [11]. As presented in Table 1 and Table 2, reducing the PPO/PEO ratio from 0.30 (F1) to 0.28 (F2) significantly (*p* < 0.0001) increased the time required for phase transition from 90 to 180 s. The blending of additive polymers slightly altered the sol-to-gel transition time. On the contrary, the inclusion of NaCl significantly (*p* < 0.0001) reduced the sol-to-gel transition time, as NaCl enhances the hydrophobic interactions among poloxamer chains, facilitating the fast transition to a 3D gel network.

### 3.2. Fourier Transform Infra-Red (FTIR) Spectroscopy

**Figure 2:**
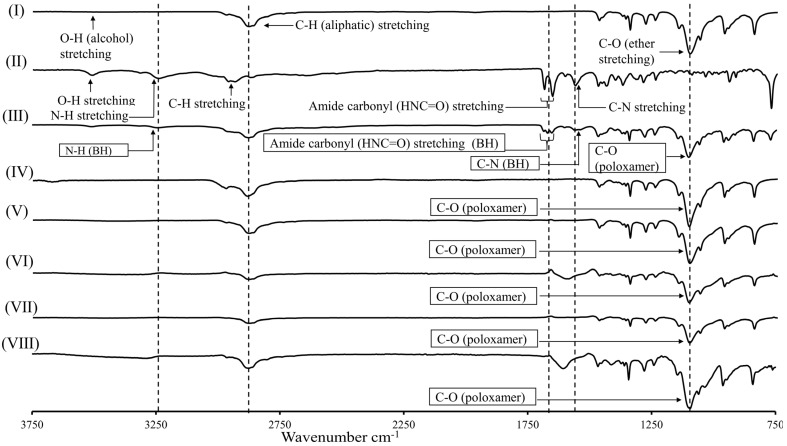
FTIR spectra of (**I**) blank F1, (**II**) bupivacaine hydrochloride (BH), (**III**) poloxamers: BH (1:10 mixture), (**IV**) blank F1NaCl, (**V**) blank F1CH, (**VI**) blank F1CMC, (**VII**) blank F1MC, and (**VIII**) blank F1SA. The vertical lines and outlined text boxes are added to guide for the shifted peaks.

FTIR analysis was performed to study the intermolecular interaction between various components of the gel matrix. As presented in Figure 2, the inclusion of additive polymers into poloxamers triggered mild intermolecular interactions, which agrees with the literature [13,37,38,39,40]. The blending of sodium alginate into poloxamer solution caused the shifting of the hydroxyl O-H (2968.3 cm^−1^) and the ether C-O-C (1099.7 cm^−1^) stretching of poloxamers to 2962.5 and 1093.6 cm^−1^, respectively. This could be attributed to the hydrogen bonding between the carboxylic and ether groups of sodium alginate with the ether and hydroxyl groups of poloxamers [37,40]. Likewise, the blending of methylcellulose, carboxymethylcellulose, and chitosan into poloxamer solutions induced shifting of its O-H stretching to 1103.3, 1105.1, and 1101.3 cm^−1^, respectively, attributed to the hydrogen bonding between additives (hydroxyl group of chitosan and methylcellulose and carboxylic group of the carboxymethylcellulose) and the ether groups on poloxamers. The hydrogen bonding between the additives and poloxamers has been reported to facilitate the physical entanglement and transition into a 3D structure, causing a reduction in the sol-to-gel transition temperature and enhancing the mechanical properties of the formed gels [13,37,38,39,40]. The blending of NaCl with poloxamer solution caused a shifting of its ethereal oxygen transmittance (C-O-C) from 1099.3 to 1103.2 cm^−1^, and C-H alkane from 1359.7 to 1348.1 cm^−1^, suggesting ionic crosslinking of sodium cation with the electronegative oxygen of poloxamer [46].

Likewise, the mixing of BH with poloxamers caused a shifting of its characteristic peaks compared to the spectrum of BH alone; N-H stretching shifted from 3240.7 to 3243.7 cm^−1^ and C-N (2nd amine) stretching shifted from 1562.2 to 1560.3 cm^−1^. Likewise, the spectrum of poloxamers showed a shift of the ether (C-O-C) absorption frequency from 1099.7 to 1101.3 cm^−1^. This might suggest a hydrogen bonding between secondary amine (NH) of BH (serves as a proton donor) and the electronegative ethereal oxygen of poloxamers [10]. The vibrational stretching of the amide carbonyl group changed from a doublet band (1687.8 and 1655.2 cm^−1^) to a triplet band (1687.3, 1672.7, and 1655.3 cm^−1^), suggesting the presence of hydrogen-bonded and non-bonded carbonyl groups within the lattice [47]. Of note, the chemical interaction between poloxamers and the loaded drug is postulated to reduce its diffusion from the gel matrix and extend its release profile [1,48].

### 3.3. Mechanical Properties

#### 3.3.1. Gel Hardness and Strength

The mechanical properties of the selected formulations are shown in Table 3. As presented, F2 demonstrated significantly lower mechanical properties compared to F1, attributed to its lower PPO/PEO ratio. Decreasing the PPO/PEO ratio reduces the hydrophobic interactions between poloxamer chains and maintains higher water content attributed to the relatively hydrophilic nature of the matrix, and therefore causes a reduction in its mechanical properties. On the contrary, blending of additive polymers significantly (*p* < 0.05) increased the gel mechanical properties compared to the base formulation F1, which could be explained by the chemical interaction between additives and poloxamers, as demonstrated by the FTIR analysis. In addition, physical entanglement between polymeric additives and poloxamers is reported to promote the gel’s mechanical properties [13,37,38,39,40]. Interestingly, F1NaCl demonstrated the highest gel strength and hardness, which could be attributed to its salting-out effect and NaCl–poloxamer interactions, as demonstrated by our FTIR findings [43]. It has been previously reported that NaCl (1% *w*/*w*) triggered a 60-fold increase in gel strength of poloxamer-based gel (15% *w*/*w* P407: 15% *w*/*w* P188) [43]. Our findings, with data not presented here, indicated that NaCl increases the gel mechanical properties as a function of its concentration (gel strength recorded as 258.8, 238.7.1, 207.0, and 124.8 at NaCl concentrations of 0.9, 0.6, 0.4, and 0% *w*/*w*), which is in agreement with the literature [42].

The gel strength and hardness might play a crucial role in defining the release profile and predicting the in vivo performance of poloxamer-based thermoresponsive gels, wherever the formulation is subject to mechanical stress such as intraarticular administration [21,49]. Formulations with low mechanical properties are likely to disintegrate rapidly, causing fast drug release and triggering toxicity issues. In comparison, formulations that exhibit adequate mechanical strength to resist deformation are likely to maintain their integrity for a longer time, offering the possibility of achieving a sustained drug release [1].

#### 3.3.2. Injectability

Table 3 shows the total work and maximum force required for injectability. In agreement with gel strength findings, the work required for F2 injectability was significantly (*p* < 0.001) smaller than F1, suggesting its easier parenteral administration attributed to its lower viscosity (see Section 3.4.1). Except for chitosan, all the additive polymers significantly promoted the work required for injection (*p* < 0.005), which is explained by the increased viscosity due to physical tangling and hydrogen bonding between poloxamers and the additives [13,37,38,39,40]. Interestingly, NaCl slightly, yet not significantly, increased the injectability force/work as compared to F1.

Injectability is a key parameter in evaluating injectable formulations, and it refers to the force/work required to expel the formulation out of a needle through a syringe [50]. It is an important parameter for characterising injectable formulations as per the FDA [31]. Formulations requiring an injectability force above 3000 g may not be suitable for parenteral administration [31,51]. The results suggest that all the developed formulations are suitable for parenteral administration. However, polymeric additives render the formulations more difficult to administer.

### 3.4. Rheological Analysis

#### 3.4.1. Frequency Sweep

Identifying the linear viscoelastic region of a thermoresponsive gel is essential for further rheological characterisation [52]. It was observed that all the formulations demonstrated linear viscoelasticity in the low strain amplitude range of 0.001% to 1%, followed by a sharp decrease in the storage modulus as amplitude strength was increased beyond 1%. Thus, the strain amplitude was kept below 1% in further rheological studies to ensure that the gel formulations maintain their internal structure and integrity.

**Figure 3:**
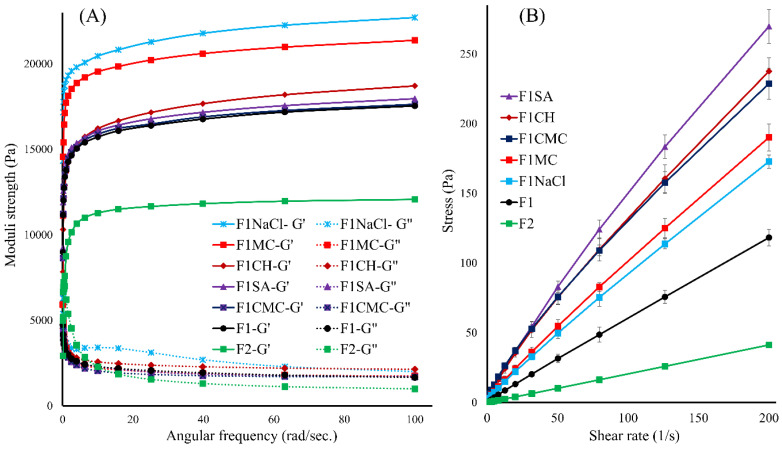
(**A**) Rheograms of the developed gel formulations at 37 °C, showing the dominant elastic properties in test conditions. (**B**) Change in shear stress as a function of shear rate at 20 °C, showing the Newtonian-like flow behaviour of the formulations.

The rheological behaviour was studied at 37 °C ± 0.5 and under deformation to predict the in vivo performance [9,53,54]. As presented in Figure 3A, all the formulations demonstrated dominant elastic properties compared to the viscous properties, independently of the angular frequency, indicating that all formulations existed in a solid-like gel status in test conditions. The results showed an apparent increase in the elastic modulus (G’):viscous modulus (G”) ratios with frequency rise, indicating an increase in the internal friction as a function of frequency. In agreement with the mechanical study findings, formulation F2 exhibited significantly (*p* < 0.001) lower elastic strength, as compared to F1, attributed to its lower PPO/PEO ratio. All the polymeric additives promoted the elastic properties of F1, which may be due to hydrogen bonding, as demonstrated in Section 3.2. In addition, the physical entanglement between poloxamers and the additive polymers might contribute to enhancing the rheological properties. Of note, F1NaCl exhibited the highest elastic strength, which could be explained by its salting-out effect, promoting the micellar dehydration, and its crosslinking with poloxamers which enhances the micellar entanglement. It is suggested that a formulation with strong elastic properties might maintain its integrity for a longer time, providing a sustained release of the encapsulated drug [54]. In contrast, a hydrogel with dominant viscous properties is likely to rapidly deform under stress, causing the fast release of the encapsulated drug, particularly where it is subject to a high applied stress such as intraarticular administration.

#### 3.4.2. Flow Sweep Analysis

The flow behaviour was determined as it directly affects the ease of administration for an injectable formulation. As presented in Figure 3B, all formulations demonstrated near Newtonian flow, with a viscosity independent of the shear rate (R^2^ > 0.995 for all formulations), suggesting their suitability for parenteral administration. Formulation F2 demonstrated significantly lower viscosity compared to F1, corroborating findings of the mechanical studies. On the other hand, all additives promoted the system viscosity, which correlates with the findings of the mechanical studies, attributed to the physical entanglement and chemical crosslinking between poloxamers and additives [13,37,38,39,40].

### 3.5. Analytical Method Development and Validation

A satisfactory peak resolution was obtained using a Kinetex C_18_ ODS (250 × 4.6 mm i.d., and 5 μm particle size) and a mobile phase potassium phosphate buffer (20 mM, pH 6.15):acetonitrile:methanol (25:35:40) and flowing at 1 mL/min (Table 4). A wavelength of 210 nm was chosen for BH quantification, as it exhibited a high UV absorbance at this wavelength. Bupivacaine exhibited a linear response between absorbance and concentration (regression coefficient (R^2^) = 0.9997) over the range of 0.5–100 µg/mL. Accuracy was satisfactory (accuracy coefficient 98.57% ± 0.5), and peak specificity/selectivity was confirmed by the peak purity test using Agilent Openlab CDS ChemStation software version 3.3.36 (Agilent, Waldbronn, Germany). The purity factor was calculated as 999.996 compared to a purity threshold of 999.993. The method demonstrated excellent repeatability (relative standard deviations of peak area and retention time were 0.44% and 0.15%, respectively) and ruggedness (relative standard deviation of the peak area within different days = 0.73%).

### 3.6. In Vitro Drug Release and Kinetic Modelling Studies

**Figure 4:**
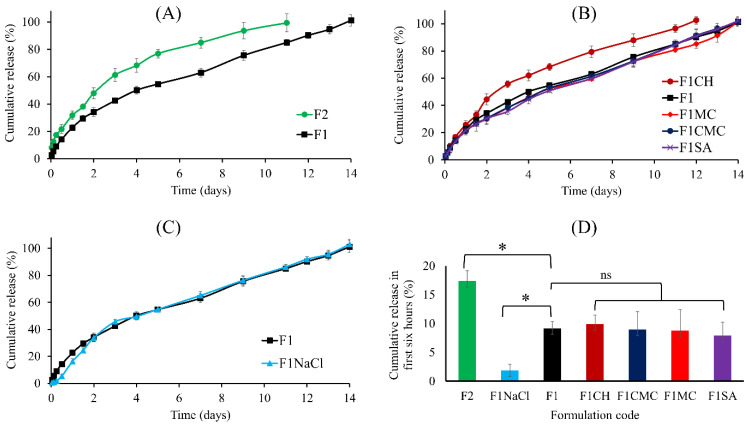
(**A**–**C**) In vitro release profile of the developed thermoresponsive gel formulations over two weeks, showing the sustained BH release (n = 3). (**D**) Percentage of BH released over the first six hours of test formulations, ns: statistically non-significant (*p* > 0.05), * statistically significant (*p* < 0.05) using ordinary one-way ANOVA, with multiple comparisons to formulation F1.

The in vitro release curves are presented in Figure 4. The base formulation F1 demonstrated sustained BH release over 14 days, with an initial burst release of 9.1% ± 1.2. This relatively long release profile [11] can be attributed to its relatively high PPO/PEO ratio and concentrated matrix, hindering gel erosion and drug diffusion. The intermolecular interactions between BH and poloxamers, as demonstrated by FTIR, may have also contributed to the extended-release profile.

Formulation F2, with a reduced PPO/PEO ratio, demonstrated a significant increase in BH release rate and a burst release over the first six hours was observed to be 17.3% ± 1.8 (Figure 4D). The physical blending of additive polymers did not significantly modify the release profile, as compared to the base formulation F1. On the contrary, the inclusion of NaCl (formulation F1NaCl) significantly reduced the burst release in the first six hours to 1.8% ± 1.1 (Figure 4D). The calculated similarity factor showed the dissimilarity between the release profiles of F1 and F2 (Table 5). On the contrary, a similarity between release profiles of the additive containing formulations and the base formulation F1 was observed.

F1 and F2 had the same total concentration of poloxamers (28.5% *w*/*w*), but an altered PPO/PEO ratio. F2 (PPO/PEO = 0.28) demonstrated a significantly (*p* < 0.0001) faster release profile, compared to F1 (PPO/PEO = 0.30). The time required to achieve 100% cumulative BH release decreased from 14 to 10 days (Figure 4A). In addition, the burst release over the first six hours increased from 9.1% ± 1.2 to 17.3% ± 1.8 (Figure 4D), as compared to the base formulation F1. This could be attributed to its longer sol-to-gel transition time, shown in Table 2. In addition, the lower PPO/PEO ratio increases the aqueous content within the gel matrix and may facilitate the bulk gel erosion and drug diffusion to the external environment.

Though the polymeric additives significantly increased the rheological and mechanical properties of the developed gel, their influence on BH release was not statistically significant (*p* > 0.05) (Figure 4B). The agitation rate during release experiments was only 10 rpm, this may have limited gel erosion and prevented the differences in mechanical properties between the formulations influencing release.

The inclusion of NaCl significantly (*p* < 0.0005) reduced the initial burst release over the first six hours, as compared to the base formulation F1 (Figure 4C,D). This could be attributed to its effects on the sol-to-gel transition as well as its effect on lattice arrangement and system porosity. As presented in Table 2, NaCl significantly reduced the sol-to-gel transition time, reducing the lag time between injection into the release medium and gel formation. In addition, we have previously demonstrated that the inclusion of NaCl into poloxamers significantly reduced the critical micellisation temperature and facilitated the system transition from a disordered to an ordered matrix [29]. Furthermore, we demonstrated that NaCl significantly reduced the intermicellar spaces and system porosity. Of note, these findings may carry significance in clinical applications, as they allow the total dose to be increased without leading to the potential systemic toxicity of BH that could be associated with its initial burst release ^1^. Of note, the overall release profile from F1NaCl was similar to F1 (f2 = 69, Table 5).

These findings and the literature data ascertain that for the formulation, scientists should critically consider the in vivo conditions and the dose volume before deciding to include NaCl in poloxamers. When comparing the contradictory results of NaCl, it is obvious that there were considerable variations in the experimental conditions. Investigating the release where the formulations (1 g) were in direct contact with the release media showed an increase in meloxicam (MW = 341.5, practically water insoluble) release when NaCl was included [21]. In contrast, injecting a large formulation volume (50 g) in a semipermeable dialysis bag demonstrated a reduction in nimesulide (MW = 308, sparingly water soluble) release with the inclusion of NaCl [14]. It is likely that the formulation volume and the surrounding environment alter the integrity of the gel and, therefore, the dissolution of NaCl into the medium, leaving a porous gel matrix behind.

### 3.7. Mathematical Modelling

To identify the mechanisms of release of BH, mathematic kinetic models were applied to the obtained release data (Table 5). As shown, the Higuchi’s model had the best fit for all formulations except F2, indicating that BH release follows Fick’s law, with diffusion as the dominant mechanism of release [8]. Generally, the release of small hydrophilic molecules from poloxamer matrices is governed by their diffusion through the interconnected aqueous channels within the gel matrix [48,55]. On the other hand, BH release from F2 was well described by the Hixson–Crowell model, indicating a considerable contribution of the gel erosion mechanism [56]. This could be attributed to its relatively hydrophilic nature, allowing the buffer diffusion into the gel matrix and facilitating its erosion.

## 4. Conclusions

This study compared three different formulation strategies to minimise the initial burst release and sustain the release of bupivacaine hydrochloride from poloxamer-based thermoresponsive gels. The findings suggest that increasing the hydrophobic component of poloxamer gels is a simple and successful approach to reduce the initial burst release and sustain the release profile. Likewise, the inclusion of NaCl is effective to limit the initial burst release. Meanwhile, the physical blending of additive polymers was not effective to sustain the release of bupivacaine hydrochloride. Modifying drug release patterns from poloxamer-based gels is achievable via simple formulation strategies.

## Figures and Tables

**Table 1:** Composition and sol-to-gel transition temperature of all the formulations trialled.

Formulation Code	Composition	Total Poloxamers Conc. (wt.%)	PPO/PEO Ratio ^†^	Sol-To-Gel Temperature (°C), Mean ± SD
F1	P407 (23%), P188 (5.5%), H_2_O (71.5%)	28.5	0.30	27.0 ± 0.00
F2	P407 (20%), P188 (8.5%), H_2_O (71.5%)	28.5	0.28	35.5 ± 0.33
F3	P407 (19%), P188 (5.5%), H_2_O (75.5%)	24.5	0.25	40.3 ± 0.47
F4	P407 (21%), P188 (5.5%), H_2_O (73.5%)	26.5	0.28	33.3 ± 0.47
F5	P407 (25%), P188 (5.5%), H_2_O (69.5%)	30.5	0.32	*
F6	P407 (27%), P188 (5.5%), H_2_O (67.5%)	33.5	0.34	*
F1SA	Sodium alginate (0.5 wt.%) + F1	28.5	0.30	26.0 ± 0.00
F1SA2	Sodium alginate (0.75 wt.%) + F1	28.5	0.30	25.0 ± 0.33
F1SA3	Sodium alginate (1.0 wt.%) + F1	28.5	0.30	23.0 ± 0.33
F1SA4	Sodium alginate (0.5 wt.%) + Calcium chloride (0.5 wt.%) + F1	28.5	0.30	**
F1CMC	Carboxy methylcellulose (0.35 wt.%) + F1	28.5	0.30	26.3 ± 0.33
F1CMC2	Carboxy methylcellulose (0.75 wt.%) + F1	28.5	0.30	23.3 ± 0.33
F1MC	Methylcellulose (0.35 wt.%) + F1	28.5	0.30	25.6 ± 0.33
F1MC2	Methylcellulose (0.5 wt.%) + F1	28.5	0.30	24.3 ± 0.67
F1MC3	Methylcellulose (0.75 wt.%) + F1	28.5	0.30	22.3 ± 0.33
F1CH ***	Chitosan (MMW) (0.4 wt.%) + F1	28.5	0.30	25.6 ± 0.33
F1CH2 ***	Chitosan (MMW) (0.5 wt.%) + F1	28.5	0.30	25.6 ± 0.33
F1CH3 ***	Chitosan (MMW) (0.75 wt.%) + F1	28.5	0.30	24.3 ± 0.33
F1NaCl	Sodium chloride (0.4 wt.%) + F1	28.5	0.30	25.4 ± 0.33
F1NaCl2	Sodium chloride (0.6 wt.%) + F1	28.5	0.30	24.3 ± 0.00
F1NaCl3 ****	Sodium chloride (0.9 wt.%) + F1	28.5	0.30	22.9 ± 0.00

*: Gelation occurred at room temperature. **: addition of calcium chloride caused precipitate formation. ***: chitosan solution was prepared in glacial acetic acid (1.5% *v*/*w*), to enhance its solubility, then mixed with poloxamer solution. ****: a white precipitate was observed at 0.9% *w*/*w* sodium chloride after storage for more than a month. ^†^: PPO/PEO ratio was calculated based on the following equation: PPO/PEO = ((P407 Conc. × 0.325) + (P188 Conc. × 0.190))/total poloxamer concentration), where all concentrations are in % *w*/*w*.

**Table 2:** Sol-to-gel transition temperature of selected BH-loaded formulations, and the time required to achieve sol-to-gel transition at 37℃. The data shows that BH inclusion increases the sol-to-gel transition temperature (n = 3).

Formulation Code	Sol-To-Gel Transition Temperature (°C) (Mean ± SD)	Sol-To-Gel Transition Time (S) (Mean ± SD)
F1	28.3 ± 0.33	90.0 ± 0.00
F2	36.3 ± 0.66	180.0 ± 4.08
F1SA	27.3 ± 0.66	81.6 ± 2.35
F1MC	26.6 ± 0.00	83.3 ± 2.35
F1CMC	27.3 ± 0.00	85.0 ± 0.00
F1CH	26.6 ± 0.33	95.0 ± 4.08
F1NaCl	25.6 ± 0.33	30.0 ± 0.00

**Table 3:** Gel mechanical properties (presented as gel hardness and strength at 37 °C ± 0.5 and injectability data at 20 °C ± 0.5), showing that decreasing PPO/PEO ratio significantly reduced the gel mechanical properties, whereas sodium chloride and polymeric additives promoted the gel mechanical properties (n = 3).

Formulation Code	Gel Hardness (g) Mean ± SD	Gel Strength (g. s) Mean ± SD	Maximum Force (g) Mean ± SD	Work Needed (g. m) Mean ± SD
F1	29.0 ± 3.4	124.8 ± 12.8	1688.0 ± 55.6	27.5 ± 1.7
F2	23.3 ± 1.1	102.3 ± 7.6	981.7 ± 32.0	15.9 ± 1.1
F1SA	40.3 ± 5.5	168.5 ± 23.0	2598.9 ± 219.8	47.4 ± 4.4
F1MC	39.8 ± 3.5	165.4 ± 13.6	2096.6 ± 90.5	37.1 ± 2.1
F1CMC	40.2 ± 2.6	166.8 ± 10.2	2526.1 ± 143.8	46.4 ± 2.8
F1CH	40.5 ± 4.6	166.2 ± 17.6	2448.1 ± 115.8	28.0 ± 1.1
F1NaCl	50.75 ± 2.7	207.0 ± 18.4	1703.6 ± 89.3	29.2 ± 2.1

**Table 4:** Optimised chromatographic conditions of the developed HPLC method.

Parameters	Details
Column	Kinetex C18 ODS, 250 × 4.6 mm i.d., and 5 μm particle size
Column temperature	Room temperature
Mobile phase	Potassium phosphate buffer (pH 6.15–20 mM):acetonitrile:methanol (25:35:40)
Flow rate	1.0 mL/min
Injection volume	10 µL
Detector	Diode array detector (DAD)
Detection wavelength	210 nm

**Table 5:** Correlation coefficients when release data were fitted to kinetic models, and calculated similarity factor (f2) of release profiles, as compared to the base formulation F1.

Formulation Code	Zero-Order	First-Order	Higuchi	Hixson–Crowell	Similarity Factor (f2)
	R^2^				
F1	0.961	0.848	0.997	0.885	NA
F2	0.897	0.980	0.989	0.991	43
F1SA	0.980	0.830	0.989	0.877	74
F1MC	0.971	0.780	0.992	0.841	74
F1CMC	0.977	0.847	0.992	0.889	80
F1CH	0.913	0.894	0.994	0.920	51
F1NaCl	0.949	0.848	0.993	0.891	69

## References

[1] Abdeltawab H., Svirskis D., Sharma M. (2020). Formulation strategies to modulate drug release from poloxamer based in situ gelling systems. Expert Opin. Drug Deliv..

[2] Bodratti A.M., Alexandridis P. (2018). Formulation of Poloxamers for Drug Delivery. J. Funct. Biomater..

[3] Haddadi H., Nazockdast E., Ghalei B., Haddadi H., Nazockdast E. (2008). Chemorheological characterization of thermosetting polyurethane formulations containing different chain extender contents. Polym. Eng. Sci..

[4] Larsen C., Østergaard J., Larsen S.W., Jensen H., Jacobsen S., Lindegaard C., Andersen P.H. (2008). Intra-articular depot formulation principles: Role in the management of postoperative pain and arthritic disorders. J. Pharm. Sci..

[5] Sosnik A., Cohn D., Román J.S., Abraham G.A. (2003). Crosslinkable PEO-PPO-PEO-based reverse thermo-responsive gels as potentially injectable materials. J. Biomater. Sci. Polym. Ed..

[6] Jeong B. (2011). Injectable biodegradable materials. Injectable Biomaterials: Science and Applications.

[7] Qiu Y., Hamilton S.K., Temenoff J. (2011). Improving mechanical properties of injectable polymers and composites. Injectable Biomaterials: Science and Applications.

[8] Akkari A.C.S., Papini J.Z.B., Garcia G.K., Franco M.K.D., Cavalcanti L., Malfatti-Gasperini A., Alkschbirs M.I., Yokaichyia F., de Paula E., Tófoli G.R. (2016). Poloxamer 407/188 binary thermosensitive hydrogels as delivery systems for infiltrative local anesthesia: Physico-chemical characterization and pharmacological evaluation. Mater. Sci. Eng. C.

[9] Ricci E.J.J., Lunardi L.O.O., Nanclares D.M.A.M.A., Marchetti J.M.M. (2005). Sustained release of lidocaine from Poloxamer 407 gels. Int. J. Pharm..

[10] Svirskis D., Chandramouli K., Bhusal P., Wu Z., Alphonso J., Chow J., Patel D., Ramakrishna R., Yeo S.J., Stowers R. (2016). Injectable thermosensitive gelling delivery system for the sustained release of lidocaine. Ther. Deliv..

[11] Sharma M., Chandramouli K., Curley L., Pontre B., Reilly K., Munro J., Hill A., Young S., Svirskis D. (2018). In vitro and ex vivo characterization of an in situ gelling formulation for sustained lidocaine release with potential use following knee arthroplasty. Drug Deliv. Transl. Res..

[12] Ummadi S., Shravani B., Rao N.R., Reddy M.S., Sanjeev B. (2013). Overview on controlled release dosage form. Int. J. Pharma. Sci..

[13] Lin H.-R.R., Sung K.C., Vong W.-J.J. (2004). In Situ Gelling of Alginate/Pluronic Solutions for Ophthalmic Delivery of Pilocarpine. Biomacromolecules.

[14] Yuan Y., Cui Y., Zhang L., Zhu H.-P., Guo Y.-S., Zhong B., Hu X., Zhang L., Wang X.-H., Chen L. (2012). Thermosensitive and mucoadhesive in situ gel based on poloxamer as new carrier for rectal administration of nimesulide. Int. J. Pharm..

[15] Zhang L., Parsons D.L., Navarre C., Kompella U.B. (2002). Development and in-vitro evaluation of sustained release Poloxamer 407 (P407) gel formulations of ceftiofur. J. Control. Release.

[16] Yu Z.-G., Geng Z.-X., Liu T.-F., Jiang F. (2015). In vitro and in vivo evaluation of an in situ forming gel system for sustained delivery of Florfenicol. J. Vet.-Pharmacol. Ther..

[17] Zaki N.M., Awad G.A., Mortada N.D., Abd Elhady S.S. (2007). Enhanced bioavailability of metoclopramide HCl by intranasal administration of a mucoadhesive in situ gel with modulated rheological and mucociliary transport properties. Eur. J. Pharm. Sci..

[18] Sridhar V., Wairkar S., Gaud R., Bajaj A., Meshram P. (2018). Brain targeted delivery of mucoadhesive thermosensitive nasal gel of selegiline hydrochloride for treatment of Parkinson’s disease. J. Drug Target..

[19] Wang W.-Y.Y., Hui P.C.L., Wat E., Ng F.S.F., Kan C.-W., Lau C.B.S., Leung P.-C. (2016). Enhanced Transdermal Permeability via Constructing the Porous Structure of Poloxamer-Based Hydrogel. Polymers.

[20] Marcos X., Pérez-Casas S., Llovo J., Concheiro A., Alvarez-Lorenzo C. (2016). Poloxamer-hydroxyethyl cellulose-α-cyclodextrin supramolecular gels for sustained release of griseofulvin. Int. J. Pharm..

[21] Inal O., Yapar E.A. (2013). Effect of Mechanical Properties on the Release of Meloxicam from Poloxamer Gel Bases. Indian J. Pharm. Sci..

[22] Juhasz J., Lenaerts V., Tan P.V.M., Ong H. (1990). Effect of sodium chloride on physical characteristics of poloxamer 407 solutions. J. Colloid Interface Sci..

[23] Park Y.-J., Yong C.S., Kim H.-M., Rhee J.-D., Oh Y.-K., Kim C.-K., Choi H.-G. (2003). Effect of sodium chloride on the release, absorption and safety of diclofenac sodium delivered by poloxamer gel. Int. J. Pharm..

[24] Liu Y., Yang F., Feng L., Yang L., Chen L., Wei G., Lu W. (2017). In vivo retention of poloxamer-based in situ hydrogels for vaginal application in mouse and rat models. Acta Pharm. Sin. B.

[25] Xuan J.-J., Balakrishnan P., Oh D.H., Yeo W.H., Park S.M., Yong C.S., Choi H.-G. (2010). Rheological characterization and in vivo evaluation of thermosensitive poloxamer-based hydrogel for intramuscular injection of piroxicam. Int. J. Pharm..

[26] Galgatte U.C., Chaudhari P.D. (2014). Preformulation study of poloxamer 407 gels: Effect of additives. Int. J. Pharm. Sci..

[27] Becker D.E., Reed K.L. (2012). Local Anesthetics: Review of Pharmacological Considerations. Anesth. Prog..

[28] Shah J.C., Maniar M. (1993). pH-Dependent solubility and dissolution of bupivacaine and its relevance to the formulation of a controlled release system. J. Control. Release.

[29] Abdeltawab H., Svirskis D., Boyd B.J., Hill A., Sharma M. (2021). Injectable thermoresponsive gels offer sustained dual release of bupivacaine hydrochloride and ketorolac tromethamine for up to two weeks. Int. J. Pharm..

[30] Jones D.S., Woolfson A.D., Brown A.F. (1997). Textural analysis and flow rheometry of novel, bioadhesive antimicrobial oral gels. Pharm. Res..

[31] Shavandi A., Bekhit A.E.-D.A., Sun Z., Ali M.A. (2016). Injectable gel from squid pen chitosan for bone tissue engineering applications. J. Sol-Gel Sci. Technol..

[32] Zhang Q., Fassihi M.A., Fassihi R. (2018). Delivery Considerations of Highly Viscous Polymeric Fluids Mimicking Concentrated Biopharmaceuticals: Assessment of Injectability via Measurement of Total Work Done “WT”. AAPS PharmSciTech.

[33] Hu J., Chen D.-W., Quan D.-Q. (2011). Rheological properties of poloxamer 407 aqueous solutions. Yao Xue Xue Bao = Acta Pharm. Sin..

[34] Guideline I.H.T. (2005). Validation of Analytical Procedure: Text and Methodology Q2 (R1). http://www.ich.org/fileadmin/Public_Web_Site/ICH_Products/Guidelines/Quality/Q2_R1/Step4/Q2_R1__Guideline.pdf.

[35] Fathalla Z.M.A., Vangala A., Longman M., Khaled K.A., Hussein A., El-Garhy O.H., Alany R.G. (2017). Poloxamer-based thermoresponsive ketorolac tromethamine in situ gel preparations: Design, characterization, toxicity and transcorneal permeation studies. Eur. J. Pharm. Biopharm..

[36] Zhang K., Shi X., Lin X., Yao C., Shen L., Feng Y. (2015). Poloxamer-based in situ hydrogels for controlled delivery of hydrophilic macromolecules after intramuscular injection in rats. Drug Deliv..

[37] Cidade M.T., Ramos D.J., Santos J., Carrelo H., Calero N., Borges J.P. (2019). Injectable Hydrogels Based on Pluronic/Water Systems Filled with Alginate Microparticles for Biomedical Applications. Material.

[38] Dewan M., Bhowmick B., Sarkar G., Rana D., Bain M.K., Bhowmik M., Chattopadhyay D. (2015). Effect of methyl cellulose on gelation behavior and drug release from poloxamer based ophthalmic formulations. Int. J. Biol. Macromol..

[39] Majithiya R.J., Ghosh P.K., Umrethia M.L., Murthy R.S.R. (2006). Thermoreversible-mucoadhesive Gel for nasal delivery of sumatriptan. AAPS PharmSciTech.

[40] Khlibsuwan R., Khunkitti W., Pongjanyakul T. (2020). Alginate-poloxamer beads for clotrimazole delivery: Molecular interactions, mechanical properties, and anticandidal activity. Int. J. Biol. Macromol..

[41] Bermudez J.M., Grau R. (2011). Thermosensitive poloxamer-based injectables as controlled drug release platforms for veterinary use: Development and in-vitro evaluation. Int. Res. J. Pharm. Pharmacol..

[42] Zeng N., Dumortier G., Maury M., Mignet N., Boudy V. (2014). Influence of additives on a thermosensitive hydrogel for buccal delivery of salbutamol: Relation between micellisation, gelation, mechanic and release properties. Int. J. Pharm..

[43] Choi H.-G., Lee M.-K., Kim M.-H., Kim C.-K. (1999). Effect of additives on the physicochemical properties of liquid suppository bases. Int. J. Pharm..

[44] de Araújo D., dos Santos A.C.M., Akkari A.C.S., Ferreira I.R.S., Páscoli M., Guilherme V.A., de Paula E., Fraceto L., de Lima R., Melo P.D.S. (2015). Poloxamer-based binary hydrogels for delivering tramadol hydrochloride: Sol-gel transition studies, dissolution-release kinetics, in vitro toxicity, and pharmacological evaluation. Int. J. Nanomed..

[45] Valero M., Dreiss C.A. (2010). Growth, Shrinking, and Breaking of Pluronic Micelles in the Presence of Drugs and/or β-Cyclodextrin, a Study by Small-Angle Neutron Scattering and Fluorescence Spectroscopy. Langmuir.

[46] Su Y.-L., Liu H.-Z., Wang J., Chen J.-Y. (2002). Study of Salt Effects on the Micellization of PEO–PPO–PEO Block Copolymer in Aqueous Solution by FTIR Spectroscopy. Langmuir.

[47] Jug M., Maestrelli F., Bragagni M., Mura P. (2010). Preparation and solid-state characterization of bupivacaine hydrochloride cyclodextrin complexes aimed for buccal delivery. J. Pharm. Biomed. Anal..

[48] Gilbert J.C., Hadgraft J., Bye A., Brookes L.G. (1986). Drug release from Pluronic F-127 gels. Int. J. Pharm..

[49] Rangabhatla A.S.L., Tantishaiyakul V., Oungbho K., Boonrat O. (2016). Fabrication of pluronic and methylcellulose for etidronate delivery and their application for osteogenesis. Int. J. Pharm..

[50] Cilurzo F., Selmin F., Minghetti P., Adami M., Bertoni E., Lauria S., Montanari L. (2011). Injectability Evaluation: An Open Issue. AAPS PharmSciTech.

[51] Burckbuchler V., Mekhloufi G., Giteau A.P., Grossiord J.L., Huille S., Agnely F. (2010). Rheological and syringeability properties of highly concentrated human polyclonal immunoglobulin solutions. Eur. J. Pharm. Biopharm..

[52] Zhu Y., Gao H., Liu W., Zou L., McClements D.J. (2020). A review of the rheological properties of dilute and concentrated food emulsions. J. Texture Stud..

[53] Mayol L., Quaglia F., Borzacchiello A., Ambrosio L., La Rotonda M.I. (2008). A novel poloxamers/hyaluronic acid in situ forming hydrogel for drug delivery: Rheological, mucoadhesive and in vitro release properties. Eur. J. Pharm. Biopharm..

[54] Baloğlu E., Karavana S.Y., Şenyiğit Z.A., Güneri T. (2011). Rheological and mechanical properties of poloxamer mixtures as a mucoadhesive gel base. Pharm. Dev. Technol..

[55] Yang L., Alexandridis P. (2000). Controlled Release from Ordered Microstructures Formed by Poloxamer Block Copolymers. Controlled Drug Delivery.

[56] Mircioiu C., Voicu V., Anuta V., Tudose A., Celia C., Paolino D., Fresta M., Sandulovici R., Mircioiu I. (2019). Mathematical Modeling of Release Kinetics from Supramolecular Drug Delivery Systems. Pharmaceutics.

